# Molecular simulation as a computational pharmaceutics tool to predict drug solubility, solubilization processes and partitioning

**DOI:** 10.1016/j.ejpb.2019.02.007

**Published:** 2019-04

**Authors:** Shakhawath Hossain, Aleksei Kabedev, Albin Parrow, Christel A.S. Bergström, Per Larsson

**Affiliations:** a Department of Pharmacy, Uppsala Biomedical Center, Uppsala University, 751 23 Uppsala, Sweden; b Swedish Drug Delivery Forum (SDDF), Uppsala University, Sweden

## Abstract

In this review we will discuss how computational methods, and in particular classical molecular dynamics simulations, can be used to calculate solubility of pharmaceutically relevant molecules and systems. To the extent possible, we focus on the non-technical details of these calculations, and try to show also the added value of a more thorough and detailed understanding of the solubilization process obtained by using computational simulations. Although the main focus is on classical molecular dynamics simulations, we also provide the reader with some insights into other computational techniques, such as the COSMO-method, and also discuss Flory-Huggins theory and solubility parameters. We hope that this review will serve as a valuable starting point for any pharmaceutical researcher, who has not yet fully explored the possibilities offered by computational approaches to solubility calculations.

## 1 Introduction

Understanding drug solubility is key to the modern drug discovery process. Achieving a sufficiently high solubility for a particular compound is absolutely crucial for processes as widely different as purification, formulation, production, drug absorption and disposition [1]. Poor solubility has many adverse effects such as reduced drug efficiency and erratic or low absorption, and may cause unwanted side effects or lack of therapeutic effect [2]. Because of this, much effort has been invested in developing tools for solubility predictions [3]. Virtual screening of solubility and solubility-related processes (e.g. dissolution, solubilization, supersaturation, precipitation) allows these properties to be evaluated before compound synthesis and is a valuable tool for computational chemists during compound library design. Computational methods that are already successfully used to calculate solubility include classical molecular simulation for solvation free energies [4], [5]. while partition coefficient calculations (log P) are used to predict solubility ratios or to provide a molecular understanding of the compound compartmentalization after solubilization [6]. Computational methods, in theory, allow a detailed understanding not only of the solubility itself, but also of the different factors and atomic level interactions that are important for the solubilization process [7], [8], [9]. In a typical pharmaceutical laboratory, however, solubility continues to be something that is determined experimentally rather than calculated or predicted, despite the feasibility of using computational methods for at least certain aspects of it, sometimes also as a more cost-efficient technique, and one which might also provide additional insights into solubilization and partitioning.

Thermodynamic equilibrium solubility is defined as the concentration at which a solid compound is in equilibrium with itself in solution [10]. Solubilization, the process leading to solvation of a solute in some solvent, is essentially a two-stage process. Solubilization requires bonds between solute molecules in their solid form to be broken, followed by formation of cavities in the solvent in which solute molecules insert themselves and interact with the surrounding solvent. Importantly, the properties of the solid solute form remain the same irrespective of the solvent in which the solubilization occurs—provided that no solvates are formed during the dissolution process. Several of the simulation methods described below make it possible to calculate relative solubility in different solvents [11], although more specialized techniques exist that also allow the solid properties to be studied [12], [13], [14]. It is worth emphasizing that even though the solid properties are often more difficult to study with simulation models, knowledge of the relative solubility of a compound in a solvent is highly warranted in itself. Knowledge of the relative solubility—combined with calculated solvation free energies and experimental knowledge of the solubility in one reference environment—pave the way to calculating absolute solubilities in any new solvent [15].

Several classes of techniques have been developed in an attempt to create a universal simulation protocol capable of solubility prediction. Most of these, such as empirical methods exploiting experimental data to analyze the contributions of specific functional groups to solubility [16], [17], and machine-learning based methods requiring training on solubility data [18], [19], are beyond of the scope of this review. Rather, our purpose here is to provide the non-experienced user an understanding of when it is possible to use molecular dynamics simulations instead of experiments to calculate solubility.

## 2 Brief introduction to molecular dynamics simulation

Since its introduction more than 40 years ago [20], molecular dynamics (MD) simulation has been used in many different research fields, and in some cases, it has become yet another tool with which a particular problem is studied. In this section we briefly summarize the main ideas behind classical MD simulations, and then present the various techniques and algorithms which the non-experienced user in a pharmaceutical laboratory can use to understand different aspects of solubility. There are already many excellent reviews on both technical and application aspects of MD [21], [22], but still, in our experience, the knowledge about what can and cannot be done with computational methods (and why that is) for studying solubility is not widespread in the pharmaceutical community. To narrow the focus further, we limit ourselves here to mainly classical, particle-based MD simulations, using any of the standard biomolecular forcefields. We also briefly compare and contrast MD with some other methods, such as Monte Carlo schemes within the grand canonical ensemble, and continuum-based models like COSMO [23], [24].

In a classical description of a system composed of discreet molecules, individual atoms move because they are experiencing forces exerted on them by all of the other atoms and molecules in the system. A critical requirement for using molecular simulation to study solubility is that the inter- and intramolecular forces in a particular system of interest can be modeled by a so-called force field, which provides the fundamental physics of the system. Within a particular force field, atoms and molecules interact primarily with each other through van der Waals’ and electrostatic forces. Additionally, within each molecule, the atoms that are covalently bound to one another are held together by strong bonded forces. In a typical biomolecular force field, all of these forces are calculated on a per-atom basis and summed up. Depending on the combination of molecule and force fields, deriving suitable interaction parameters (through the process of parameterization) for a novel molecule can be more or less straightforward, but in all cases is a process that requires careful validation. Different force field will differ in the philosophy behind the parameterization of the force field (including what sort of validation work becomes necessary for a novel molecule of interest), i.e. the determination of default values for bond lengths, van der Waals’ parameters and partial charges, to give a few examples.

The force field is what defines the physical interactions between atoms and molecules in the simulation, or computational experiment. The other major component of the experiment makes use of the forces that act between the atoms to make them move. This is achieved using numerical methods to solve Newton’s equations of motion. The forces are obtained in practice by taking the gradient of the interaction potentials involving each atom. With this procedure, it is possible to obtain the position of each of the atoms in the system at a small time-step later; from these new positions, new forces are again calculated and used to update positions a second time, and so on. The time-evolution of the entire system can then by tracked by connecting each state to the previous one. There are a number of software packages that can be used for these types of simulations, each with its merits and limitations. The more common ones include Chemistry at HARvard Molecular Mechanics (CHARMM) [25], [26], Amber [27], NAMD [28], [29], GROningen Machine for Chemical Simulations (Gromacs) [30], [31], ACEMD [32], and Desmond [33].

The connection between a discreet molecular description of a particular system (such as the study of a particular single drug molecule solvated in a surrounding solvent), and macroscopic observations on the same system in a traditional experimental setting is provided by the branch of physics called statistical mechanics. While the details of this connection are beyond the scope of this review, we will discuss it briefly on a case-by-case base in the coming sections. We will describe a number of ways in which classical simulation methods have been used to calculate and predict solubility, and to what extent these results agree with experimental solubility measurements.

## 3 Solubility

In what follows, we are primarily concerned with solubility of a solute in a solvent in the so-called infinite-dilution or solubility limit, i.e. the solvent is in such excess that the properties of the solute do not change with a change in the amount of solvent. Working at this limit computationally means that the solubility process can be simulated without considering any solute-solute interactions. Typically what is studied is either the transfer of a solute between: (i) a gas and liquid phase (free energy of solvation); (ii) gas and water phases (free energy of hydration); or (iii) two liquid phases. In terms of free energy, solubility can be expressed as the change in e.g. Gibbs free energy of transfer of solute i between two phases, α and β, using the equation:

(1) $$\Delta G_{i}^{\alpha \beta }\left(T,p,X^{\alpha },X^{\beta }\right)=-RTlnK_{i}^{\alpha \beta }$$

where X is the composition at equilibrium, R the gas constant, p pressure, and T temperature. The partition coefficient K is defined as the ratio of the number densities of solute i and phases α and β, respectively. This is also what is typically measured experimentally in solubility determinations. Naively, this would suggest that the easiest way to compare an experimentally determined solubility with a simulation is to compare the values of K obtained from the two methods. However, a direct calculation of K from simulations is prohibitive in most cases, for various reasons; for example, it may not be possible to properly sample the solute in the two different phases to acquire enough statistics for conclusions to be reliably drawn. Different techniques have therefore been developed to calculate the solubility in terms of the transfer free energy ($\Delta G_{i}^{\alpha \beta })$ instead. Equilibrium solubility requires the chemical potential μ of all species to be the same, in addition to the pressure and temperature [34], [35]. Through Henry’s law, the solubility is related to the excess, or residual, chemical potential $\mu_{i}^{E}$ [36], and in the limit of infinite dilution equals the free energy calculated from computational simulations. The word excess refers to the difference in chemical potential between that of a real and ideal system at the same temperature and pressure [37].

## 4 Free energy calculations

Four possible schemes are often presented for calculating free energy differences from simulations. These are: (i) methods based on histograms [38]; (ii) non-equilibrium simulations [39]; (iii) perturbation theory [40], [41]; and (iv) using derivatives of the potential [42]. As free energy is a path-independent state variable, one can design simulations in a computer experiment that are a convenient, albeit somewhat artificial, way to compute the free energy. This computer approach simplifies calculations and allows one to circumvent the possibly difficult task of computationally simulating the corresponding real chemical process. The unphysical nature of such simulated transitions is why some of these free energy calculation methods are referred to as alchemical. For details we refer the reader to any of the more in-depth texts on the matter [43], [44], [45], [46], but as a point of entry we note that the difference in free energy, here denoted as ΔF, between two states i and j, can be expressed in terms of the so-called partition function Z as in Eq. (2):

(2) $$\Delta F_{\mathrm{ij}}=-k_{B}T\ln\left(\frac{Z_{j}}{Z_{i}}\right)$$

where $k_{B}$ is the Boltzmann constant, T is the temperature, and Z_i_ and Z_j_ are the statistical mechanics partition functions of the two states of interest. While in principle these partition functions would provide a complete description of a system, often they are not known, which is the reason that absolute free energies of real systems are difficult to obtain from computer simulations. In terms of drug solubility these two states could, for example, correspond to an active pharmaceutical molecule being present or absent from a simulation box filled with solvent molecules.

### 4.1 Free energy perturbation calculations

**Fig. 1:**
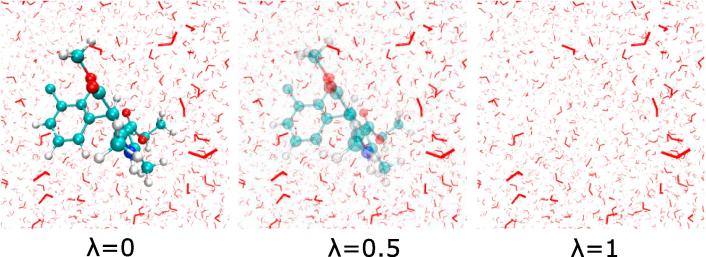
Free energy perturbation calculation in which a drug molecule (felodipine) is being gradually decoupled from the surrounding solvent molecules. The coupling parameter lambda, describes the graduale (but artificial) de-coupling of the system in a step-wise process going from lambda = 0 (felodipine is fully interacting with its surroundings) to lambda = 1, in which all drug-solvent interactions are set to zero.

Originally proposed by Zwanzig in 1954 [40], the free energy perturbation (FEP) method can be used to describe the free energy difference between two different systems. These systems correspond to the initial system i, which is unperturbed, and j, which is called the perturbed system in relation to system i. The perturbed system may for example include an additional particle or a different energy function, as would be the case when using FEP to calculate solvation free energies. The free energy difference between these two states is then related according to Eq. (3):

(3) $$\Delta F_{\mathrm{ij}}=-k_{B}Tln\left(e^{-\frac{E_{j}-E_{i}}{k_{B}T}}\right)$$

where k_B_ is Boltzmann's constant, T is temperature, and E_i_ and E_j_ are the energies associated with two different states of the system (Fig. 1).

The FEP method requires that the difference in energy between states i and j is sufficiently small, in order for the calculations to converge, and to provide a meaningful result. As a result, in practice it is normally necessary to divide a FEP calculation into a series of individual simulations in a piecewise manner through a coupling parameter lambda (Fig. 1), and these simulations are then computed independently [47], [48]. Luckily, communication between such simulations is not necessary, so the process can be trivially parallelized by running each part on a different processor/node, making calculations more efficient. Another situation in which FEP can be beneficial is when the final state's phase space is a subset of the reference state [49], [50].

### 4.2 Thermodynamic integration

An alternative to evaluating the difference in the free energy between subsequent states is to calculate the derivative of the total energy (the so-called Hamiltonian, including both potential and kinetic) in the system. Thermodynamic integration (TI) was originally proposed by Kirkwood in 1935 and is still one of the most widely used free energy calculation approaches. The TI method covers a transition between an initial and a final state, and similarly to FEP uses a coupling parameter λ to describe the transition between these two points. By introducing several intermediate λ values between 0 and 1, the energy function as a function of λ would be defined as in Eq. (4):

(4) $$U\left(\lambda \right)=U_{i}+\lambda \left(U_{f}-U_{i}\right)$$

**Fig. 2:**
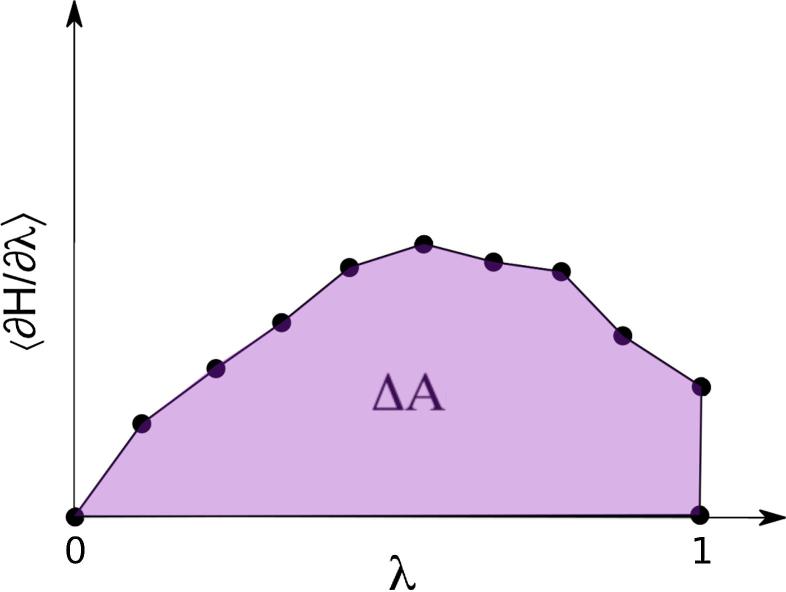
Schematic representation of the thermodynamic integration (TI) method. Here H stands for the Hamiltonian of the system in a particular moment, δH/δλ is its derivative with respect to λ (the coupling parameter, describing the extent to which the system is in state 0 or 1, and ΔA, the area under the curve, is the free energy difference between the initial and final states.

The potential energy $U\left(\lambda \right)$ in each λ -state can be calculated as an ensemble average over configurations sampled from a MD simulation. The change in free energy between the initial and final states 0 and 1 can then be computed from the integral of the ensemble-averaged derivatives of potential energy over the coupling parameter λ in Eq. (5) [22]:

(5) $$\Delta F\left(i\to f\right)=\int_{0}^{1}\langle \frac{\delta U(\lambda )}{\delta \lambda }\rangle d\lambda$$

where ΔF is the free energy and U(λ) the potential energy expressed as a function of the coupling parameter λ (Fig. 2).

### 4.3 Applications

These free energy methods have been used extensively for calculation of solvation and hydration free energies as well as chemical potential calculations in different systems, and what will follow is not meant to be an exhaustive enumeration of all such work [51], [52], [53], [54], [55]. As a first pharmaceutically relevant example, Lüder et al. [56] used FEP to calculate the free energy of solvation of 46 different drugs in pure melt systems, and reached an average error of 1.8 kJ/mol over their entire dataset. Further, using TI, the hydration free energies for some polyphenol compounds (a class of bioactive natural compounds with pharmaceutically interesting properties) such as toluene and (+)-catchecin, was calculated at different temperatures in a recent study by Gillet et al. [57]. Even though the actual hydration free energies differed (−47.78 kJ/mol calculated for toluene vs −3.22 kJ/mol as the measured value, a difference the authors attribute to differences in pressure and the water model they used), the obtained relative free energies at different temperatures showed consistent trends. The authors were also able to fit exponential functions to their results describing how solubility was moderated by temperature. These fitted functions had parameters that was found to be dependent on the properties of the solid solutes.

In a series of papers [56], [58], [59], Kjellander et al. studied the efficiency of FEP methods for the calculation of transfer free energies [58], first for drug molecules in TIP4P water using the OPLS-AA [60] force field, and then, as mentioned above, followed up by free energy of solvation in pure melts [56], and finally in amorphous matter [59]. In their simulations, the Coulomb and Lennard-Jones (LJ) interactions where scaled down independently. Interestingly, the authors also complemented the use of FEP with analysis based on using molecular energies and molecular surface areas to further understand the separate stages (cavity formation, solute insertion, dispersion interactions between solute and solvent) of the solubilization process.

Another study using FEP, work of Paluch et al. [61] illustrates how one can express the equilibrium mole fraction solubility via solvation free energy calculations and fugacity. As also suggested by Mobley and co-workers [11], the relative solubility of an active pharmaceutical ingredient in different solvents can be calculated from chemical potentials. These calculations had a root-mean-squared deviation (RMSD) of between 1.17 and 3.46 between calculated and experimental relative solubility values, depending on the method used. The advantage with such an approach is that the contribution to solubility from the fugacity of the solute in its solid form cancels out when the relative difference for the same solute in two different solvents is sought. The work from Mobley et al. shows that results from particle-based simulations agree better with experimental data than those from other methods like UNIFAC [16] (a semi-empirical method). The same approach was also used to calculate the excess solubility (solubility in an actual solution relative to what would be observed if the solvents mixed to form an ideal solution) [62] for acetanilide, acetaminophen, phenacetin, benzocaine and caffein in mixtures of water and ethanol [37], with overall excellent quantitative agreement with experiment.

## 5 Widom insertion method

Widom [38] showed that the residual or excess chemical potential in the canonical ensemble, in which the number of particles in the system is kept constant, can be expressed as in Eq. (6):

(6) $$\mu_{\mathrm{ex}}=-k_{B}T\ln{\langle e^{-\varphi /kT}\rangle }_{N}$$

where k_B_ is the Boltzmann constant, T is the temperature of the system, φ is the interaction energy of a randomly placed test particle with the system of N particles, and 〈….….〉_N_ denotes ensemble averaging over all configurations of the particles in the volume V [38], [63].

**Fig. 3:**
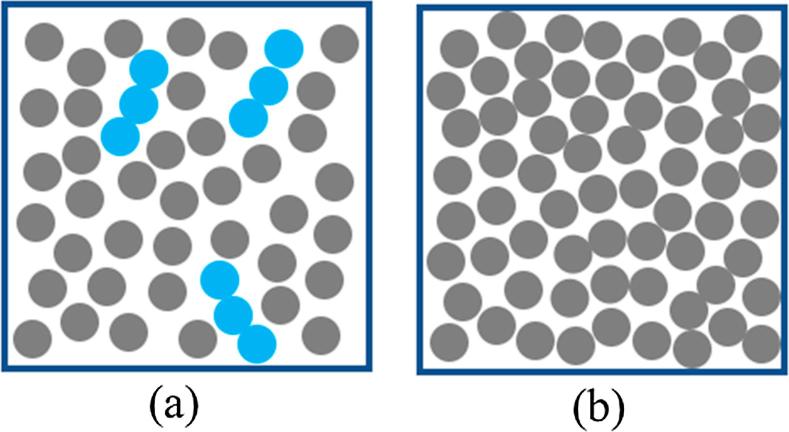
Illustration of the Widom test particle insertion method. Solvent particles are gray and molecules are blue. (a) A loosely dense system in which the molecules can be inserted in a region with cavities, (b) A highly dense system in which the molecules are unable to insert by themselves. (For interpretation of the references to colour in this figure legend, the reader is referred to the web version of this article.)

Widom’s test particle insertion scheme has been used to determine the solubility of small molecules in different systems with good agreement with experimental results in both cases [64], [65]. However, due to the required random insertion of particles (Fig. 3), overlap often occurs between atoms of the inserted solutes and the rest of the system [66], [67]. This issue becomes critical in a dense solvent or during the insertion of large molecules and can lead to errors in the calculated chemical potential (and therefore also the solubility) if adequate sampling is not performed. To alleviate this problem, Khawaja et al. [67] used a biased insertion technique, in which the molecules or particles are inserted into a pre-identified free space, and used this to determine the solubility of small molecules such as helium, carbon dioxide, and water in nitrile butadiene rubber. Their results were in good agreement with experimental solubility values and this biased insertion technique is 40 times faster than unbiased, brute-force particle insertion. To solve the same issue for the insertion of long polymeric chains, an incremental insertion technique can be implemented in which smaller parts of the solute is added sequentially to the end of one of the chains [68]. Ferguson et al. [66] studied the solubility of alkane chains up to eicosane $\left(C_{20}\right)$ in water with this technique and found good agreement with the experimental results up to pentadecane $\left(C_{15}\right)$.

Further examples that are relevant in a pharmaceutical context is the application of the Widom insertion method to predict solubility of water in polylactide (PLA) polymers [69]. The authors obtain a value of the solubility parameter (more details below) of 20.6 MPa^1/2^, which compares favorably to experimentally determined values of 19–20.5 MPa^1/2^ [70]. Even though the solute is water and not for example a drug molecule this showcases the usefulness of the Widom method. The solubility of water in PLA is important since it in turn can impact the stability of amorphous drug formulations, and again, advances of the insertion technique such as incremental insertion [66] makes it also potentially suitable for investigating the solubility of larger molecules in other dense media.

## 6 Solubility calculations in the grand canonical ensemble

The grand canonical ensemble (GCE) is the name of a thermodynamic system (or ensemble) in which the temperature and chemical potential of the species of interest are specified for a fixed volume, and the number of particles and energy are the variables [71]. The system reaches equilibrium when a number of successful insertion or deletion of particles balance each other. In this way, the GCE more closely mimics real systems in which the exchange of energy and particles occur in a regular manner. Note that in a typical canonical ensemble, the number of particles in a system is kept constant. Simulations in the grand canonical ensemble have been used to calculate chemical potential or to investigate fluid phase equilibria [72], [73], [74].

MD-simulations, but also Monte Carlo (MC)-methods, can be used to perform simulations in the GCE. Monte Carlo methods are arguably naturally better suited for this since the insertion or deletion of molecules in the GCE resembles the stochastic movements of molecules in MC-simulations [72]. The Monte Carlo method in the GCE has been used for calculating for example phase equilibria [73], [75] and the solubility of small penetrants in some flexible chains [71]. The combination of MC and the GCE, the so-called grand canonical Monte Carlo (GCMC) scheme, is often combined with a histogram-reweighting method for phase equilibrium calculations [75], [76]. Here, data on the frequency of energy and particle numbers are organized as histograms [75]. Thereafter, the free energy of a system over a range of thermodynamic conditions is determined from a limited number of simulations. Potoff et al. [75] used GCMC to obtain the phase behavior of a variety of polar and non-polar binary mixtures and determined their phase diagrams with high accuracy. Liu et al. L [77] used histogram-reweighted GCMC to obtain the phase behavior of CO_2_-H_2_0 mixtures over a broad range of temperature and pressures. They found that the existing atomistic models for water or $CO_{2}$ cannot reproduce the experimental data over the entire temperature and pressure range which indicates the importance of improving the current models. Density-biased GCMC simulations was used by Rodgers et al. to investigate the solubility of alcohol molecules in a coarse-grained lipid bilayer model [78]. The model quantitatively determined the partitioning coefficient as a function of alcohol concentration in the system. However, the authors concluded that in order to capture the partitioning over a range of temperatures improved coarse-grained models are required.

Similarly to the Widom insertion method, application of GCMC to high-density systems is difficult mainly due to the substantially lower probability of finding a large enough cavity for particle insertion or sufficiently high energy configurations to delete molecules [72], [74], [79]. Mezei et al. [80] developed a cavity–based GCMC scheme which was later modified by Yau et al. [71] This works well for moderately dense systems, but the success rate of insertion or deletion of molecules is still somewhat low.

Instead of using MC, grand canonical ensemble molecular dynamics simulation (GCMD) techniques have also been developed based on an extended Hamiltonian system in which the physical system is linked to a particle bath to represent the particle number as a continuous dynamic extension variable [81], [82], [83]. This was then successfully used to determine the solubility of gases [79] and water [74] in different polymers. An approach that uses both the GCMC and MD techniques to study the solute behavior and solubility in explicit solvent aqueous systems and a solvated protein environment was presented by Lakkaraju et al. [84]. In this method, $\mu_{\mathrm{ex}}$ of both the solute and the water systematically oscillated over the GCMC-MD iterations to drive the solute and water energy and particle exchanges. The method is capable of approximating the hydration free energy of individual organic solutes, dilute aqueous mixtures of multiple solutes, and binding affinity of solutes to the protein.

Due to the conceptual appeal to mimic systems in a realistic way, the grand canonical ensemble can be a suitable technique to calculate solubility of relevant molecules in pharmaceutical research, in particular when combined with adaptive resolution simulation schemes [85], which allows one to focus on a specific region in space at high resolution, while the rest of the system can be treated at lower resolution [85], [86]. This multi-scale approach can improve the computational efficiency when simulating large-scale systems.

## 7 Free energy methods for calculation of molecule partitioning

**Fig. 4:**
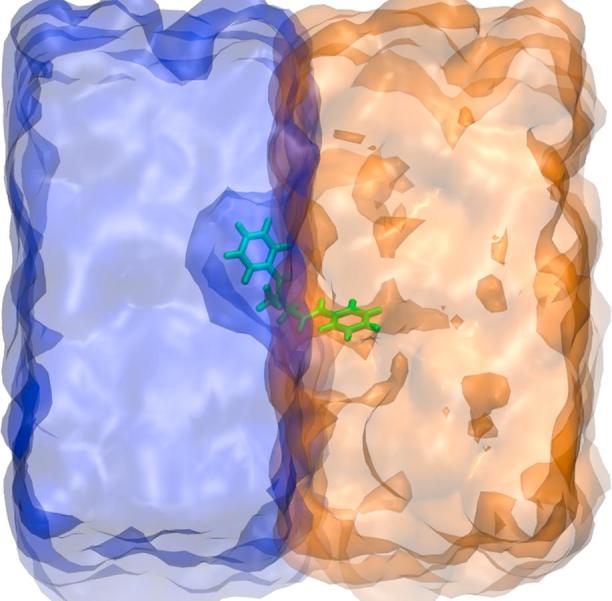
Schematic representation of the partition coefficient as the equilibrium concentrations of a dissolved substance in a two-phase system with two largely immiscible solvents (in this case water, blue surface, and hexadecane, orange surface). (For interpretation of the references to colour in this figure legend, the reader is referred to the web version of this article.)

The partition coefficient, P, is the ratio of the concentration of a neutral or unionized solute molecule in a system of two immiscible solvents (Fig. 4). It is typically expressed as the logarithm of this concentration ratio, as in Eq. (7).

(7) $$\log P=log\left(\frac{{\left[solute\right]}_{\mathrm{Organic}}}{{\left[solute\right]}_{\mathrm{water}}}\right)$$

In drug design strategies, a correct understanding of the interactions of a particular solute in both organic (lipophilic) and water (hydrophilic) phases is necessary [87], and can sometimes be just as critical as knowledge of the actual solubility. Therefore, the partition coefficient—which describes the difference in solubility of the solute in the organic and water phases—is important; it is for example used in drug design as a measure of the hydrophobicity of a solute, membrane permeability, as well as the bioavailability of drug compounds in different environments [6], [88]. When computing the partition coefficient with simulations, oils, chloroform or alkanes can be used as the organic phase, but 1-octanol is commonly used due to its amphiphilic nature (a polar head group attached to a nonpolar tail). This renders the molecule similar to the main constituents of lipid membranes and gives it the capability of mimicking different properties of a real biological system [88].

Because of the practical importance of partition coefficients, many experimental techniques have been developed to measure them [89], [90]. However, these experimental measurements use actual compounds which can be costly and the synthesis of the compounds also often takes a long time [88]. Therefore, to accelerate the drug design and discovery process, many theoretical and computational models have been developed to determine the partition coefficient. Quantitative structure property relationship (QSPR) models can relate the log P value with different descriptors or molecular properties such as molecular surface, volume, weight, functional groups, dipole moment, etc. [91]. Additive fragment-based techniques and machine learning algorithms are also used to calculate the partition coefficient [6]. However, these methods vary in accuracy and efficiency and are all required to be trained on experimental data sets [6], [92]. As an alternative, alchemical free-energy calculation techniques (for example using FEP or TI) with molecular dynamics simulation, can be used to calculate the partition coefficient, since it is directly proportional to the transfer free energy between two solvents [6], [88], [91], [92]: The relationship between the partition coefficient and transfer free energy can be expressed as in Eq. (8):

(8) $$\log P=\frac{{-\Delta G}_{\mathrm{transfer}}}{2.303RT}=\frac{{\Delta G}_{o}-{\Delta G}_{w}}{2.303RT}$$

where R is the molar Boltzmann constant, T is the temperature, $and{\Delta G}_{o}$ and ${\Delta G}_{w}$ are the free energies in an organic solvent e.g. 1-octanol and water, respectively. This approach provides reliable predictions of the partition coefficient and can be applied to many combinations or organic solvent, water and solute. In addition, MD simulation provides molecular level insights about the partition mechanism of the solutes into the solvents.

A large number of different studies have calculated partition coefficients from different free energy calculation techniques. Best et al. e [93] used the FEP method for some small organic molecules in a saturated octanol-water system. The logP values were generally in good agreement with the experimental values except for acetamide-acetone. DeBolt and Kollman [94] successfully calculated the logP of benzene and phenol in an octanol–water system using the FEP method with modified force fields. They also investigated the structural and thermodynamic behavior of liquid octanol and postulated that octanol’s capability to serve as a biophase analog is mainly due to the formation of preferentially polar versus nonpolar regions in the media.

One of the issues of using free energy methods is that the calculated free energies are relative to a reference solute, which in turn produces a partition coefficient relative to that of the reference solute [91]. Therefore, to determine the absolute partition coefficient, an experimental partition coefficient is required for the reference solute. An accurate molecular model or force field is also required for both the reference solute and solute of interest. Garrido et al. [95] found excellent agreement (average deviations of 0.2, 1.1, 0.8 and 1.2 kJ mol^−1^) between experimental and simulated logP values for propane, benzene, ethanol and acetone respectively through the calculation of the free energies of solvation using MD simulation and thermodynamic integration. The absolute average deviation between the experiments and simulation was 0.28 logP units. However, this deviation could be lowered to 0.14 logP units by optimizing partial atomic charges of acetone in the water phase. Huang et al. [96] reported predicted logP values for different solute classes including alkanes, chlorides, bromides, fluorides, compounds with sulfur, phosphorus etc. They used an implicit solvation model in which 3D-RISM [97] theory was used to calculate the solvation free energy for different solute classes including alkanes, chlorides, compounds with sulphur, phosphorous etc. They obtained a good prediction of logP compared to the experimental values. Ogata et al. [88] calculated the octanol–water logP for 75 compounds, including 17 drug compounds, using free energy calculation with the Bennett’s acceptance ratio (BAR) [41] method. For the set of 17 drug molecules in particular, the agreement obtained between experimental and computational logP values had an R^2^ value of 0.86 and a mean squared error (MAE) for the solvation free energies of 2.26 kJ/mol. Using the BAR method, Wolf and Groenhof [98] calculated hydration free energies and chloroform–water partition coefficients for different nucleic acids, molecules which are similar in size and complexity to many pharmaceutical drugs. They found, at least for their dataset, that the CHARMM [25] force fields reproduced the experimental data more accurately (absolute error of 0.65 log P units) than other force fields i.e. AMBER [27], GROMOS [99], and OPLS-AA [60]. These studies clearly indicate the importance of an accurate force field or molecular model all molecules in the system before using free energy and MD simulation techniques to calculate the logP values of the molecules.

Adding further to the plethora of different kind of computational schemes, Bhatnagar et al. used an adaptive biasing force method with MD simulations (ABF-MD) to calculate the partition coefficients of n-alkanes. One of the major advantages of this method is that it does not need reference solutes. The so-called Gibbs ensemble Monte Carlo (GEMC) is another method which can calculate the transfer free energies and determine the partition coefficients directly by simulating two phases simultaneously [100], [101]. In this scheme, molecules can transfer directly between the two phases during the simulation and the free energy of transfer can be calculated directly from the number density of particles in the two phases, according to Eq. (9) [102]:

(9) $${\Delta G}_{\mathrm{transfer}}=2.303RTlog\left(\frac{\rho_{\alpha }}{\rho_{\beta }}\right)$$

where $\rho_{\alpha }$ and $\rho_{\beta }$ represent the number density of particles in the two α and β, respectively. This method works well for small molecules for which transfer of solute molecules occurs between phases at a reasonable rate.

In two other studies, the partition coefficient for 150 [103] and 650 [104] small compounds, respectively, was also predicted by free energy calculation performed with coarse-grained MD (CGMD) simulations using the MARTINI [105] force-field. CGMD reduces the simulation time compared to all-atom MD and was found capable of reproducing the experimental octanol–water and hexane–water partition coefficients (in one of the studies [103] the deviations was between 0.67 and 0.90 log P units). Some discrepancies for the computed solvation free energies (mean absolute deviations larger than 10 kJ/mol [103]) where mainly attributed to the limited fluid range of the Lennard-Jones potential in the MARTINI coarse grained force field. Implementation of a hybrid all-atom/coarse-grained model unfortunately failed to improve the discrepancy for the estimation of solvation free energy [103].

It should be noted that to estimate the transfer free energy, ${\Delta G}_{\mathrm{transfer}}$ in Eq. (9), the solvents of the system are generally assumed to be completely immiscible and the solvation free energy of each solute is calculated separately. However, in theory, very polar solutes can carry water molecules to the organic phase [6], complicating the picture. Furthermore, as noted in the introduction, during free energy calculations only one solute molecule is present in the solvent, so that the calculation can be considered to take place at infinite dilution. During experiments, significant deviation of the solute concentration from infinite dilution can lead to differences between experimental and computational values. Other errors inherent to different experimental methods can be another reason for the discrepancies in some studies between the measurements and simulations. However, from the studies discussed above, it is evident that free energy methods with suitable force fields and enough sampling can predict partition coefficients accurately and efficiently; this is advantageous for rapid screening of large numbers of drug compounds.

Switching gears a bit, yet another method to calculate log P is the Conductor-like Screening Model for Realistic Solvation (COSMO-RS) [23]; which extended to micelles and membranes is called COSMOmic [24]. This latter approach allows efficient prediction of partition coefficients of molecules in micelles and lipid bilayers. The details regarding COSMO-RS and COSMOmic can be found in [23], [24], [106]. Combinations of COSMOmic and MD methods [106] have been used to calculate the logP of cytochrome P450 substrates and their metabolites [107], 4-ethylphenol, propanol, 5-phenylvaleric acid, and dibenz[a,h]anthracene [108], and other drug-like molecules [109] in various model membranes: dioleoylphosphatidylcholine (DOPC), palmitoyloleoyl-phophatidylglycerol (POPG), 1-stearoyl-2-oleoyl-*sn*-glycero-3-phosphocholine (SOPC), and 1-palmitoyl- 2-oleoyl-*sn*-glycero-3-phosphocholine (POPC). Paloncyova et al. [112] also compared the partition coefficients and thermodynamic properties obtained by COSMOmic calculation and MD simulation and there was good agreement between the two methods. The COSMOmic calculation is faster than the MD simulations. However, MD simulations can be more informative for estimating the membrane localization of drug-like molecules and for analyzing structural features of complex systems, e.g. lipids, drugs and proteins [112].

Finally, the umbrella sampling (US) method can also be used to calculate free energy profiles (potentials of mean force), and from them log P [110], [109], [112]. Paloncyova et al. [111] compared the logP obtained using DOPC and POPG bilayers with the octanol–water logP and found that the octanol–water logP correlates with only some types of membranes. This suggests that the typical organic–water system logP cannot be straightforwardly used for all types of membranes and molecules. Another study by Holmboe et al. [110] compared the influence on the partition coefficient of danazo, felodipine and carbamazepine on membrane composition, in particular the influence of bile salt insertion in membranes.

## 8 Flory-Huggins theory and solubility parameters

Initially developed as a method to study the behavior of dilute polymer solutions [111], Flory-Huggins (FH) theory has more recently been used together with MD simulations to study not only qualitative miscibility but also solubility in a number of pharmaceutically relevant systems [112], [113], [114], [115]. FH-theory, originally based on the concept of entropy of mixing of two differently sized polymers, gives a way of estimating the change in free energy upon mixing of different molecules. Closely connected to FH-theory is also the so-called Hansen-Hildebrand (HH) solubility parameters [116]. Initially devised by Hildebrand, and then later extended and refined by Hansen, these parameters, which can be calculated from simulations using the FH-machinery, can be used to predict the solubility of a pharmaceutical compound in a solution. The HH-solubility parameters can be defined as the square root of the so-called cohesive energy density [117] of the (amorphous) system at room temperature. The cohesive energy density corresponds to, and can be measured experimentally from, the enthalpy change upon evaporation [118]. Such measurements however can be more or less complicated depending on the possibility to evaporate a particular material [119].

Computationally, the cohesive energy density is typically calculated as the difference between the non-bonded energy in the condensed and gas phases [120], [121], and from this the solubility parameter δ can then be calculated according to Eq. (10):

(10) $$\delta =\sqrt{E_{\mathrm{coh}}/V}=\sqrt{E_{\mathrm{vac}}-E_{\mathrm{bulk}}/V}=\sqrt{\mathrm{CED}}$$

where E_coh_ is the cohesive energy density, E_vac_ and E_bulk_ the potential energies of molecules in vacuum and the condensed phases respectively, and V system volume. Depending on whether the Hansen or Hildebrand solubility parameters are sought, the non-bonded energies can be further split to reflect contributions from different types of interactions. As mentioned by Choi et al. [122], care has to be taken when calculating the gas phase energies, since polymer conformations in this phase most likely differs from those in the condensed phase. A protocol for this has been published by Belmares et al. [123] among others.

Having calculated the CED, it is then possible to calculate the also the FH interaction parameter $X_{\mathrm{FH}}$, which describes the miscibility of the system components, using Eqs. (11), (12) [120], [121].

(11) $$\Delta E_{\mathrm{mix}}=\phi_{1}{\left(\frac{E_{\mathrm{coh}}}{V}\right)}_{1}+\phi_{2}{\left(\frac{E_{\mathrm{coh}}}{V}\right)}_{2}-{\left(\frac{E_{\mathrm{coh}}}{V}\right)}_{1,2}$$

(12) $$\chi_{\mathrm{FH}}=\frac{V}{\mathrm{RT}}{\Delta E}_{\mathrm{mix}}$$

E_coh_ again the cohesive energy density as in Eq. (10), Φ refers to the molar fractions of the respective molecules in the system, V is volume, R the gas constant and T temperature.

In this way, the miscibility has been studied for a number of different systems using classical MD by many researchers. Perhaps due to its origin within polymer theory, the vast majority of these systems involve polymers in some way, and either with or without the addition of small molecules such as drugs. Studies on polymer-polymer blends include those of Yang et al. [124], who calculated the solubility parameters and miscibility of poly-3-hydroxybutyrate (PHB) and polyethylene oxide (PEO) using the Dreiding force field [125]. Their results were in agreement with literature reference values. PEO was also used in a study by Chaudari et al. [126] in which they describe the free energy change of mixing PEO in water. Prathab et al. [8] investigated interfacial interactions between polymethyl methacrylate and a number of other polymers with the COMPASS [127] forcefield to establish favorable interactions in their mixed systems. Another polymer-polymer mixing study of PEO together with polyvinyl chloride, and concluded that 70/30 or 30/70 blends are more miscible than a 50/50 blend [128]. The COMPASS forcefield was again used by de Arenaza el al. [129] to study poly-L-lactide and poly-D-lactide with polystyrene and PVP; they found that the calculated values deviated 10–20% from the measured ones. The temperature dependence on solubilty can also be addressed using the cohesive energy density concept, as was done for example by Chen et al. [130], who modeled the glass–rubber transition for amorphous polymers.

Studies on systems including additional, non-polymeric molecules, include that of Gupta et al. [131] who compared experimental and computational results for solubility of indomethacin in combination with either PEO, glucose or sucrose using the COMPASS [127] force field. Xiang and Anderson [9] similarly performed simulations of indomethacin in poly-vinylpyrrolidone (PVP). Force-field parameters for these compounds were derived to be compatible with the Amber family of forcefields, and the FH-interaction parameter calculated from their simulations predicted miscibility between PVP and indomethacin at molar fractions in agreement with experimental results. Hyunh et al. [120] again used the COMPASS force field for MD simulations of solubilty calculations of docetaxel, an anti-cancer agent, in a number of excipients (tricaprylin, tricaproin, tributyrin, vitamin E and beta-carophyllene). As can be shown from FH-theory. using a miscibility cutoff value of less than 0.5 for the FH interaction parameter and varying the fractions of drug and excipients, they were able to determine solubility values for their systems in agreement to within 2–6% of experiments. The combination of small molecules (4-*n*-pentyl-4_-cyanobiphenyl (5CB)) and polymer polymethyl methacrylate (PMMA) was also investigated by Patnaik and Pachter [132]. Using FH theory, they concluded that while 5CB would mix with monomeric methyl methacrylate, it is not miscible with PMMA. Xiang and Anderson [133] calculated FH interaction parameters for mixtures of felodipine, hydroxypropylmethylcellulose (HPMC) and water, and the value of the FH-interaction parameter (−0.20 + −0.07) predicted miscibility of felodipine and HPMC at all HPMC compositions. The simulations were also able to predict a disrupting effect of water on the hydrogen bonds between felodipine and HPMC.

An interesting alternative to the use of cohesive energy density for calculating solubility parameters was recently published by Callaway et al. [134] They proposed instead an interaction energy based on the molecular volume enclosed by a Connolly surface. They note that this leads to a more realistic description of the interaction energy between two molecules, which might otherwise fail to take into account the combination of molecular size and affinity strength. Wu and co-authors devised an entirely new force field (TEAM) to validate predictions of solubility parameters for polymers of different chain lengths. They found that their computed solubility parameters were progressively underestimated with increasing polymer length [135].

## 9 Equations of state parameters from molecular simulation

Another route to predict drug solubility from simulations it to combine them with an equation of state (EOS). The most prominent example of an EOS is arguably the ideal gas law, which relates pressure and volume to the absolute temperature and the number of moles of the gas. A number of studies have used such a combined EOS-MD approach to calculate the solubility of different compounds. For example, Zhong and Masuoka [136] used the Peng-Robinson EOS to calculate gas solubilities in molten polymers. Using the Sanchez-Lacombe equation, Gauter el al. [137] predicted isotherms of ethylene and n-hexane in PE. Also using the Sanchez-Lacombe equation, Kiparissides et al. [138] determined the solubility of ethylene and found good agreement with measurements. Other efforts to this end include publications by Sato et al. [139], [140], [141], [142], Kroon et al. [143], and Lisal et al. [144]

The common theme for these studies is that they require the determination of a number of thermodynamic state variables before any modeling of, or any conclusions about, the solubility. This is something that might not be straightforward, and in some cases even involve additional modeling or parameter fitting. Computational modeling can be helpful in such cases, as many of the relevant parameters can be estimated using simulations instead. This was illustrated in a study by Kanellopoulos et al. [145] who again employed the Sanchez-Lacombe equation, but this time using simulations with the COMPASS forcefield to determine pressure, temperature and density, and hence solubility of α-olefins in polyolefins. Fermeglia and Pricl [146] showed, for two different EOS, how to use molecular simulation to extract characteristic parameters for four synthetic polymers.

## 10 Conclusions

Solubility is at the heart of pharmaceutical research. Understanding it is crucial for any research project, whether within academia or in industry. The purpose of this review has been to outline the various ways in which computational modeling, in particular molecular simulation, can be used to calculate and predict solubility. We have tried to showcase examples where simulations can guide or even preempt experiments. The increase in computational power allows more and more complex systems to be studied. In particular small-molecule simulations have matured from a tool for single solute investigations into something that allows a large number of compounds to be rapidly screened for their compatibility with a particular solvent. This can be done just as efficiently as the corresponding screen in the traditional laboratory, but also faster and arguably also at a lower cost. In addition, and perhaps just as importantly, a molecular level description of solubilization might provide further insights into the underlying mechanisms and processes.
